# The interaction between lifestyle and blood pressure on Stroke: A cross-sectional study from Northern China

**DOI:** 10.1371/journal.pone.0344016

**Published:** 2026-03-09

**Authors:** Jiantao Yu, Qiang Zhou, Yanyan Zhao, Haiying Chen, Huiyong Yu, Yuying Li, Xiuguo Zhang

**Affiliations:** 1 Hebei Medical, University Second Hospital, Shijiazhuang, China; 2 Hebei Medical, University Third Hospital, Shijiazhuang, China; 3 College of Nursing, Hebei Medical University, Shijiazhuang, China; University of Cape Town, SOUTH AFRICA

## Abstract

**Objective:**

This study aimed to explore and quantify the extent of interaction between lifestyle factors and systolic and diastolic blood pressure on stroke among adults over 65 years of age. This investigation sought to provide valuable insights into the multifactorial risk factors for stroke, with the ultimate goal of supporting clinicians in implementing more focused and comprehensive preventive strategies.

**Methods:**

Data were obtained from the 2019 health examination records of community hospitals in northern China. A stratified cluster random sampling method was used to select a representative sample from resident health records within the essential public health service management system. Participants were categorized into four subgroups based on systolic and diastolic blood pressure levels. Odds ratios (ORs) with 95% confidence intervals (CIs) and trend tests were used to examine the association between blood pressure categories and incident stroke. In the lifestyle subgroup analysis, a multiplicative interaction model within binary logistic regression was employed to assess the effect of interactions between unhealthy lifestyles and different blood pressure levels on stroke.

**Results:**

A total of 34,995 eligible subjects were included in the analysis, comprising 44.3% males (n = 15,484) and 55.7% females (n = 19,511). The age range was 65–103 years, with a mean age of 71.91 ± 5.65 years. After adjusting for confounding factors, systolic and diastolic blood pressure (both as continuous and categorical variables) showed a linear positive correlation with stroke incidence(Ptrend for SBP = 0.001; Ptrend for DBP = 0.001). In the lifestyle subgroup analysis, this positive correlation remained significant across most subgroups; Furthermore, Interaction and joint effect analyses revealed that elevated SBP/DBP and unhealthy lifestyle factors synergistically augmented stroke risk after multivariable adjustment.

**Conclusions:**

This study demonstrated that elevated systolic and diastolic blood pressure were significantly associated with a higher incidence of stroke. A synergistic effect was observed between unhealthy lifestyle factors (such as smoking, alcohol consumption, obesity, and physical inactivity) and elevated blood pressure in increasing stroke risk. Smoking partially mediated the relationship between diastolic blood pressure and stroke. These findings highlight the potential benefit of integrated control strategies targeting both blood pressure and modifiable lifestyle factors.

## Introduction

Stroke is a group of cerebrovascular diseases characterized by acute brain tissue injury resulting from the sudden rupture or obstruction of cerebral blood vessels. It is associated with high morbidity, high mortality, and high disability rates, placing a substantial economic burden on patients and their families. As a major global health issue, stroke ranks as the third-leading cause of both death and disability worldwide [1]. According to the latest Global Burden of Disease Study (GBD), the lifetime risk of stroke in China is 39.9% [2], the highest in the world, and is increasing at an annual rate of 8.3% [3]. Therefore, the prevention and treatment of stroke represent an urgent public health challenge that requires immediate attention.

The etiology of stroke is complex and influenced by a combination of individual, genetic, behavioral, and environmental factors [2,4–6]. Understanding its multifactorial nature is essential for enhancing prevention and treatment strategies. Findings from the “INTERSTROKE” study across 32 countries indicated that modifiable risk factors—including hypertension, dyslipidemia, obesity, smoking, and alcohol consumption—accounted for 90.7% of the population-attributable risk of stroke [7]. Among these, hypertension remains one of the most significant modifiable risk factors and is globally prevalent. Epidemiological data show that approximately 73.91% of stroke patients have comorbid hypertension [6]. A synthesis of 61 prospective observational studies worldwide (involving approximately one million individuals aged 40–89 years) demonstrated a continuous, independent, and direct positive association between clinic-measured systolic blood pressure (SBP) and diastolic blood pressure (DBP) and stroke-related mortality. For blood pressure levels ranging from 115/75 mmHg to 185/115 mmHg, each increase of 20 mmHg in SBP or 10 mmHg in DBP was associated with a doubling of the risk of cardiovascular and cerebrovascular events [8]. However, the effects of systolic and diastolic blood pressure on stroke risk among older adults remain controversial. A study by Miura et al. conducted among middle-aged and elderly individuals in Japan reported a positive correlation between both SBP and DBP and stroke risk [9]. In contrast, Makino et al. found that in hypertensive patients over 70 years of age, stroke risk was not associated with elevated systolic blood pressure [10]. Some studies have even suggested that lower blood pressure may increase stroke risk, possibly due to reduced cerebral perfusion leading to hypoperfusion and hypoxia, which could subsequently contribute to incident stroke [11].

However, in recent years, a growing number of researchers have focused on the significant role of modifiable factors, particularly lifestyle, in reducing the risk of stroke. The American Heart Association/American Stroke Association (AHA/ASA) has issued guidelines for the secondary prevention of stroke, which advocate for health-promoting behaviors such as reducing cigarette and alcohol consumption and increasing physical activity. These recommendations aim to decrease stroke-related morbidity and mortality among stroke survivors by controlling risk factors and encouraging the adoption of healthy behaviors [12].

Although existing research has extensively examined the individual associations between hypertension and lifestyle with stroke, a significant gap remains regarding their interactive or joint effects on stroke risk. Furthermore, the underlying mechanisms are not fully understood, and current studies lack robust causal inferences pertaining to the combined influence of lifestyle and blood pressure on stroke. There is a clear need to investigate the interaction between these factors. Such research would enhance our understanding of stroke etiology, improve risk stratification, and potentially contribute to the development of updated treatment guidelines that account for multiple interacting risk factors. This study aims to provide valuable insights into the multifactorial risk profile of stroke, with the ultimate goal of supporting clinicians in delivering more targeted and comprehensive preventive interventions.

## Methods

### Research object and data source

To promote comprehensive health, the Chinese Ministry of Health established primary healthcare institutions to provide free health management services—including annual health examinations for adults aged 65 and older—and to create electronic health records for this population. This retrospective cross-sectional study was conducted within the framework of this long-term chronic disease management project for older adults in northern China. We utilized data collected during the screening period from February to September 2019. A stratified cluster random sampling method was employed to obtain a representative sample from the resident health records within the Essential Public Health Service Management System. All participants provided written informed consent allowing the use of their medical records for research purposes. All data were fully anonymized prior to access and analysis.

The study design and sampling procedure were conducted as follows. Participants were selected from all 11 cities within a province in northern China. In the first stage, two subdistricts or townships from both urban and rural areas were selected from each city using probability proportional to size sampling, based on the population of each subdistrict/township. In the second stage, two additional subdistricts were chosen from the previously selected districts by simple random sampling. Finally, two community hospitals were randomly selected from each subdistrict. All residents aged 65 years or older who voluntarily participated in the health examination at the selected community hospitals were included in the study. The inclusion criteria were: (1) age ≥ 65 years, and (2) residency in the target county or district for more than six months. The exclusion criteria included: (1) hearing impairment or inability to communicate normally. (2) inability to cooperate due to cognitive impairment or mental illness. A total of 39,179 individuals aged over 65 years from the selected communities were initially enrolled. After excluding participants with missing values in the lifestyle survey, 34,995 individuals were included in the final analysis, yielding a response rate of 89.32%. All covariates with complete data were incorporated into the study.

### General study questionnaire

An interview-based survey was conducted by trained staff using a standardized questionnaire. Sociodemographic data were collected, including sex, age, occupation (laborer, peasant, or leadership), educational level (college graduate, high school graduate, or below high school), marital status (married, never married, or widowed/divorced), medical insurance status, family history of stroke, and medical history (including atrial fibrillation, hypertension, and diabetes mellitus). Lifestyle information was obtained through self-reporting via a structured questionnaire, covering details such as smoking habits, alcohol consumption, and physical activity. Smoking status was categorized into three groups: never smoker, former smoker, and current smoker. Current smokers were defined as those who smoked at least one cigarette per day for a minimum of six months. Drinkers were defined as individuals who consumed at least 30 mL of alcohol per week for one year or longer. Non-exercisers were identified as participants who had not engaged in leisure-time or recreational physical activity for at least one year, and who had not performed medium or heavy manual labor during that period. It should be noted that transport-related physical activity was not assessed in this survey.

### Anthropometric tests and laboratory examinations

Qualified medical professionals conducted physical and laboratory examinations. Participants were instructed to remove their shoes and wear lightweight clothing for the measurement of weight and height. An automatic sphygmomanometer was used to measure blood pressure (BP). Measurements were performed twice in a quiet environment, with participants resting for at least 10 minutes between each measurement. If the difference between the two readings exceeded 5 mmHg, a third measurement was taken, and the mean of all measurements was calculated as the final blood pressure value. Fasting blood samples were collected in the morning after an overnight fast. These samples were used to assess fasting blood glucose (FBG), triglycerides (TG), total cholesterol (TC), low-density lipoprotein cholesterol (LDL-C), and high-density lipoprotein cholesterol (HDL-C). Additionally, imaging examinations included abdominal color ultrasound, chest radiography, and electrocardiography.

### Ascertainment of Stroke

The identification of stroke cases in this study was based on a combination of self-reported medical history and neurologist assessment, in accordance with World Health Organization criteria. Additional information regarding symptoms, date of onset, diagnostic units, medical records, and imaging data were collected to validate the initial diagnosis. Stroke was defined as an event comprising subarachnoid hemorrhage, intracerebral hemorrhage, or cerebral ischemic necrosis. Cases of secondary stroke due to transient cerebral ischemia, brain tumor, metastatic brain tumor, or trauma were excluded.

### Definition of independent variables

Hypertension was diagnosed in patients with systolic blood pressure (SBP) ≥140 mmHg or diastolic blood pressure (DBP) ≥90 mmHg in the absence of antihypertensive medication, as well as in those with a documented history of hypertension who were receiving antihypertensive treatment, irrespective of current blood pressure measurements [13]. According to the evidence-based guidelines for adult hypertension in China, hypertension was classified into four categories [14]: Normotension (SBP < 140 mmHg and DBP < 90 mmHg), Grade 1 hypertension (SBP 140–159 mmHg and/or DBP 90–99 mmHg), Grade 2 hypertension (SBP 160–179 mmHg and/or DBP 100–109 mmHg), and Grade 3 hypertension (SBP ≥ 180 mmHg and/or DBP ≥ 110 mmHg). Based on these criteria, SBP and DBP were each divided into four groups: Q1 (SBP ≤ 139 mmHg), Q2 (SBP 140–159 mmHg), Q3 (SBP 160–179 mmHg), Q4 (SBP ≥ 180 mmHg); M1 (DBP ≤ 89 mmHg), M2 (DBP 90–99 mmHg), M3 (DBP 100–109 mmHg), M4 (DBP ≥ 110 mmHg). Diabetes mellitus was defined as fasting blood glucose (FBG) ≥7.0 mmol/L and/or the use of hypoglycemic medication or a previous diagnosis of diabetes [15]. Hyperlipidemia was defined as total cholesterol (TC) ≥6.2 mmol/L, low-density lipoprotein cholesterol (LDL-C) ≥4.1 mmol/L, triglycerides (TG) ≥2.3 mmol/L, or high-density lipoprotein cholesterol (HDL-C) <1.0 mmol/L [16]. Atrial fibrillation was identified based on self-reported history or findings from on-site electrocardiogram examinations. Body mass index (BMI) was calculated as weight in kilograms divided by the square of height in meters. Overweight was defined as BMI between 24.0 and 27.9 kg/m², and obesity as BMI ≥ 28.0 kg/m² [17].

### Statistical analysis

All statistical analyses were performed using SPSS 26.0 (IBM SPSS Statistics for Windows, Version 26.0. Armonk, NY: IBM Corp). A two-sided P value <0.05 was considered statistically significant. Descriptive analyses were conducted across blood pressure groups. Categorical variables are expressed as numbers and percentages. Three logistic regression models were fitted to estimate odds ratios (ORs) and their corresponding 95% confidence intervals (CIs) for the association between blood pressure and stroke. Trend tests were performed using the median value of each systolic blood pressure (SBP) and diastolic blood pressure (DBP) group. Subgroup analyses were carried out to evaluate the effects of both continuous and categorical SBP and DBP on stroke incidence across various lifestyle subgroups, including obesity (yes/no), smoking (yes/no), drinking (yes/no), and exercise (yes/no). Multiplicative interaction terms within logistic regression models were used to examine the interaction effects between lifestyle factors and different SBP/DBP groups on stroke.

### Statement of Ethics

The research complied with the ethical guidelines outlined in the Declaration of Helsinki and received approval from the Ethics Committee of The Hebei Medical University Third Affiliated Hospital China. All participants are informed about the study and written informed consent. Furthermore, all collected data are kept confidential and anonymous.

## Results

### General characteristics of study subjects

A total of 34,995 individuals were included in the analysis, comprising 44.3% males (n = 15,484) and 55.7% females (n = 19,511). The age range was 65–103 years, with a mean age of 71.91 ± 5.65 years. Among the participants, 924 (2.6%) were identified as stroke patients. Hypertension was the most prevalent chronic condition, affecting 58.4% of participants, followed by hyperlipidemia (31.4%), diabetes (23.3%), and fatty liver (21.3%). Unhealthy lifestyle factors included smoking (11.5%), alcohol consumption (10.3%), obesity (14.1%), and physical inactivity (60.0%) (Supplementary File, S1 Table).

### Association of systolic and diastolic blood pressure with Stroke

Tables 1 and 2 present the associations between SBP and DBP(both as continuous and categorical variables) and the incidence of stroke. After adjusting for confounding factors (including sex, age, occupation, marital status, education level, medical insurance, atrial fibrillation, family history of stroke, diabetes mellitus, dyslipidemia, and fatty liver), logistic regression analysis indicated that higher levels of SBP and DBP were associated with an increased incidence of stroke. Both systolic and diastolic blood pressure showed a positive correlation with stroke incidence, and these associations were linear (Ptrend for SBP = 0.001; Ptrend for DBP = 0.001). Compared with the Q1 group of SBP, the adjusted odds ratios (ORs) with 95% confidence intervals (CIs) for the Q2–Q4 groups were 1.086 (0.926–1.274), 1.384 (1.098–1.744), and 1.648 (1.175–2.313), respectively. Compared with the M1 group of DBP, the adjusted ORs (95% CIs) for the M2–M4 groups were 0.69 (0.53–0.88), 0.52 (0.39–0.70), and 0.42 (0.30–0.58), respectively. When analyzed as continuous variables, each 1 mmHg increase in SBP and DBP was associated with a multivariable-adjusted odds ratio for stroke of OR = 1.008 (95% CI: 1.004–1.012) and OR = 1.012 (95% CI: 1.005–1.019), respectively.

**Table 1:** Multivariate-adjusted Odds ratios (95% confidence intervals) of estimated SBP for Stroke.

Systolic pressure, mmHg						
	Q1 < 140	Q2(140–159)	Q3(160–179)	Q4 ≥ 180	*P*_*trend*_	Each 1 kg/m2 increase
Total(N)	23760	7825	2493	917		
Event (n)	591	211	85	37		
OR(95%CI)						
Model 1	1	1.086(0.926-1.274)	1.384(1.098-1.744)	1.648(1.175-2.313)	0.001	1.008(1.005-1.012)
Model 2	1	1.085(0.925-1.272)	1.390(1.103-1.752)	1.652(1.177-2.319)	0.001	1.008(1.005-1.012)
Model 3	1	1.064(0.906-1.249)	1.350(1.070-1.704)	1.594(1.133-2.242)	0.001	1.008(1.004-1.011)

Model 1: No adjustment for confounding factors.

Model 2: Adjusted gender and age.

Model 3: Adjusted for sex, age, occupation, marital status, education level, medical insurance, atrial fibrillation, family history of Stroke, diabetes, dyslipidemia, and fatty liver.

**Table 2:** Multivariate-adjusted Odds ratios (95% confidence intervals) of estimated DBP for Stroke.

Diastolic pressure, mmHg						
	M1 ≤ 89	M2(90–99)	M3(100–109)	M4 ≥ 110	*P*_*trend*_	Each 1 kg/m^2^increase
Total (N)	29770	3946	990	289		
Event (n)	768	1105	37	14		
OR (95% CI)						
Model 1	1	1.032(0.840-1.269)	1.466(1.048-2.052)	1.922(1.118-3.305)	0.007	1.013(1.006-1.020)
Model 2	1	1.029(0.837-1.265)	1.444(1.031-2.021)	1.875(1.090-3.225)	0.010	1.012(1.005-1.019)
Model 3	1	1.016(0.825-1.251)	1.462(1.043-2.051)	1.828(1.060-3.155)	0.012	1.012(1.005-1.019)

Model 1: No adjustment for confounding factors.

Model 2: Adjusted gender age.

Model 3: Adjusted for sex, age, occupation, marital status, education level, medical insurance, atrial fibrillation, family history of stroke, diabetes, dyslipidemia, and fatty liver.

### Interaction effects between systolic and diastolic pressure and lifestyle on stroke

**Fig 1:**
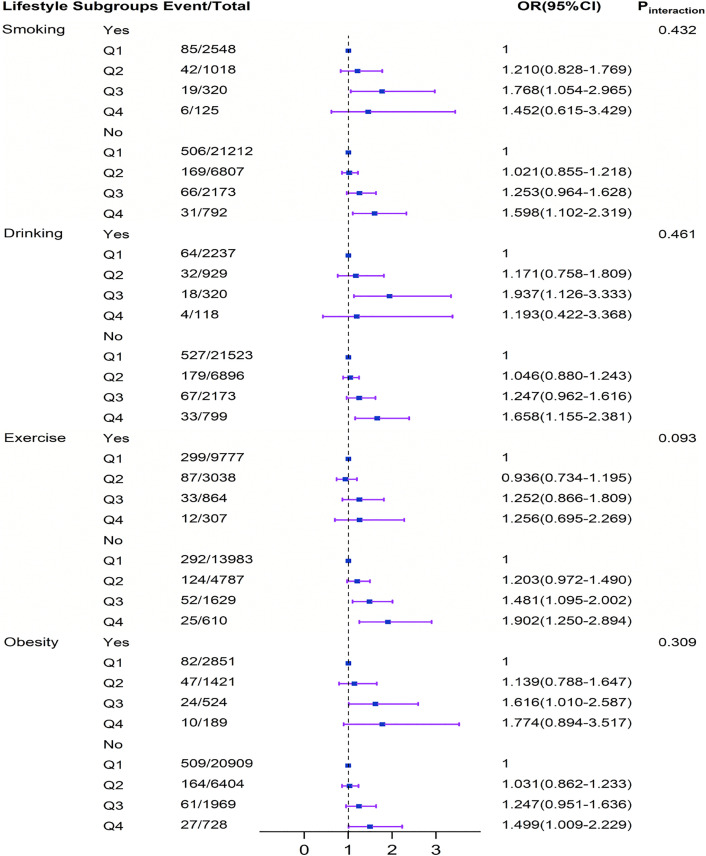
Subgroup and interaction analyses among the group Q1 − 4 of SBP and Stroke across various lifestyle subgroups. The multiplicative interaction model of Logistic regression is used to explore the impact of the interaction between lifestyle and different groups of SBP with Stroke. All models were adjusted for sex, age, occupation, marital status, education level, medical insurance, atrial fibrillation, family history of stroke, diabetes, dyslipidemia, and fatty liver. CI, conﬁdence interval; OR, odds ratio; SBP, systole blood pressure. Q1: SBP ≤ 139 mmHg, Q2: 140 ≤ SBP ≤ 159 mmHg, Q3: 160 ≤ SBP ≤ 179 mmHg, Q4: SBP ≥ 180 mmHg.

**Fig 2:**
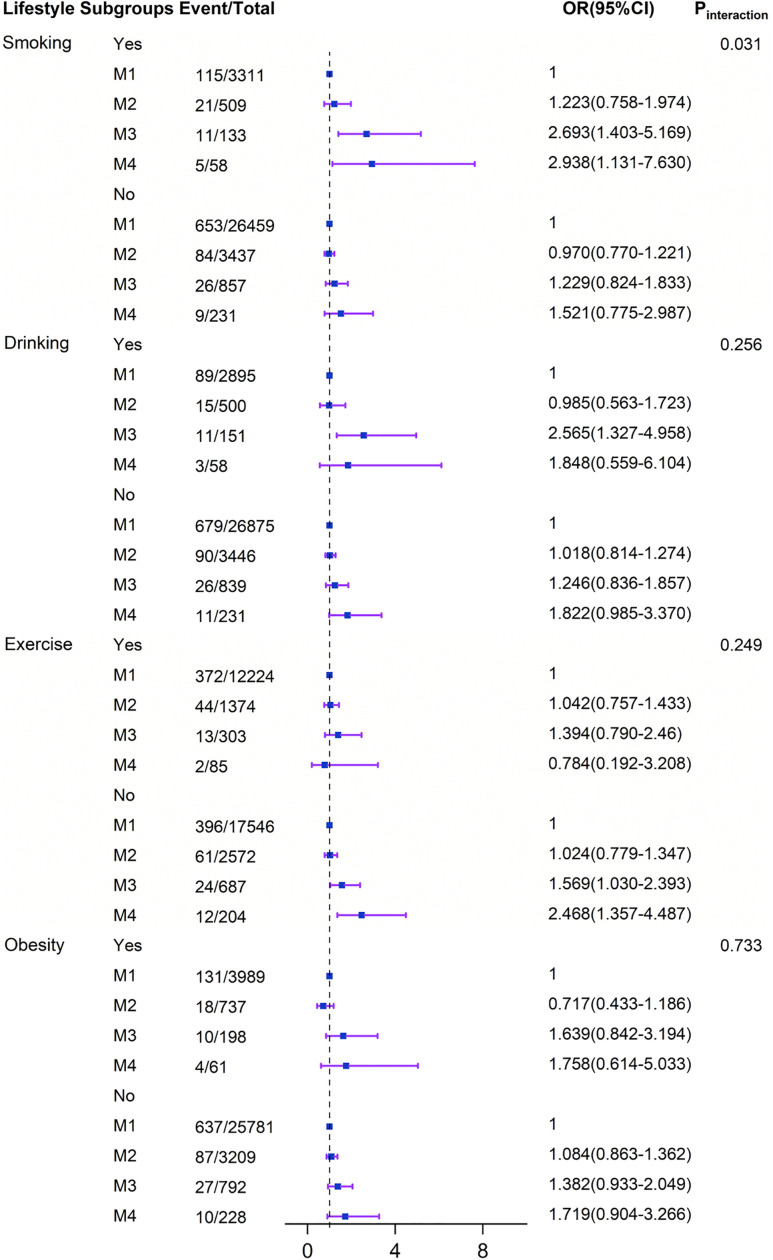
Subgroup and interaction analyses among the group M 1 − 4 of DBP and Stroke across various lifestyle subgroups. The multiplicative interaction model of Logistic regression is used to explore the impact of the interaction between lifestyle and different groups of DBP with Stroke. All models were adjusted for sex, age, occupation, marital status, education level, medical insurance, atrial fibrillation, family history of stroke, diabetes, dyslipidemia, and fatty liver. CI, conﬁdence interval; DBP, diastolic blood pressure; OR, oddsratio; M1: DBP ≤ 89 mmHg, M2: 90 ≤ DBP ≤ 99 mmHg, M3: 100 ≤ DBP ≤ 109 mmHg, M4: DBP ≥ 110 mmHg.

Stratified analyses were conducted to evaluate whether the associations between elevated systolic and diastolic blood pressure (analyzed both as continuous and categorical variables) and the incidence of stroke were modified by predefined lifestyle subgroups. The relationship between increased systolic and diastolic blood pressure and stroke incidence remained consistent with the primary results across most subgroups (Table 3; Figs 1–2). In subgroup analyses, a significant positive association between SBP and stroke incidence was observed across various lifestyle categories, including smokers, non-smokers, non-drinkers, physically inactive individuals, and those with or without obesity (all Ptrend < 0.05; see Supplementary S3 Table). Similarly, a significant positive association between DBP and stroke incidence was confirmed in subgroups such as smokers, drinkers, physically inactive individuals, and non-obese participants (all Ptrend < 0.05; see Supplementary S2 Table).

**Table 3:** Subgroup analysis of the association between estimated Systolic and diastolic blood pressure and Stroke.

		Systolic pressure, mmHg		Diastolic pressure, mmHg	
Lifestyle	N	OR(95%Cl)	P_interaction_	OR(95%Cl)	P_interaction_
Smoking			0.548		0.026
yes	4011	1.010(1.002-1.019)		1.029(1.013-1.044)	
no	30984	1.007(1.003-1.011)		1.008(1.000-1.015)	
Drinking			0.787		0.492
yes	3604	1.009(0.999-1.018)		1.019(1.001-1.037)	
no	31391	1.008(1.004-1.011)		1.011(1.003-1.018)	
Exercise			0.106		0.518
yes	13986	1.004(0.999-1.010)		1.01(0.999-1.021)	
no	21009	1.01(1.006-1.0150)		1.014(1.005-1.023)	
Obesity			0.248		0.916
yes	4985	1.011(1.003-1.019)		1.012(0.996-1.027)	
no	30010	1.006(1.002-1.010)		1.011(1.003-1.019)	

*SBP and DBP are examined as a continuous variable.

The model adjusted for sex, age, occupation, marital status, education level, medical insurance, atrial fibrillation, family history of stroke, diabetes, dyslipidemia, and fatty liver.

**Fig 3:**
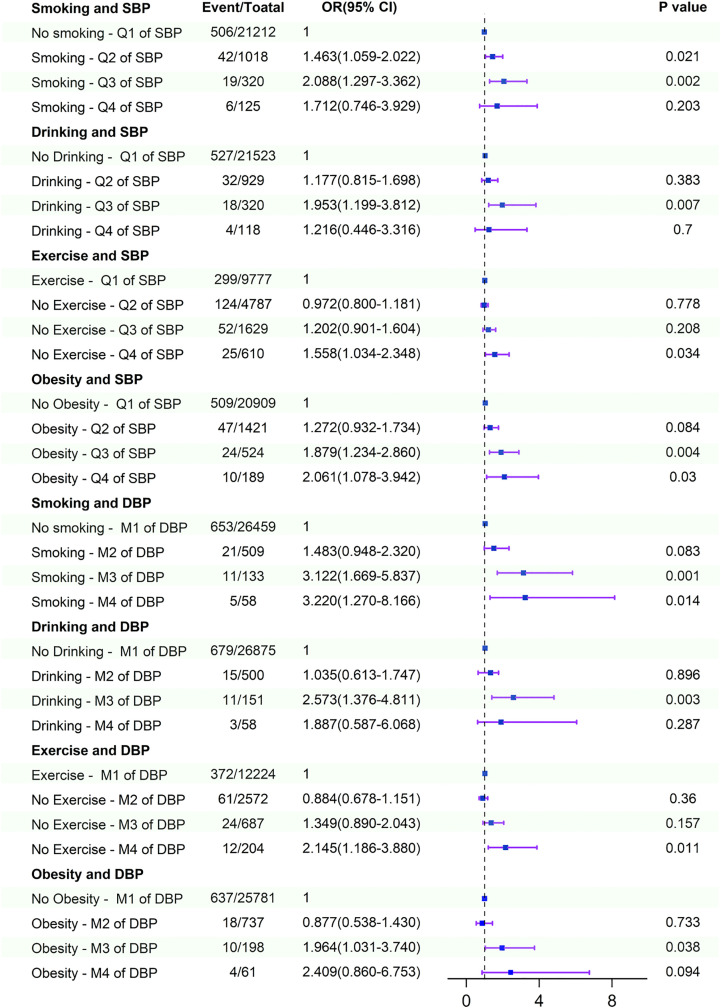
Interaction and joint effects for exposures to higher SBP and DBP and unhealthy lifestyle on Stroke. All models were adjusted for sex, age, occupation, marital status, education level, medical insurance, atrial fibrillation, family history of stroke, diabetes, dyslipidemia, and fatty liver. Q1 of SBP: SBP ≤ 139 mmHg, Q2 of SBP: 140 ≤ SBP ≤ 159 mmHg, Q3 of SBP: 160 ≤ SBP ≤ 179 mmHg, Q4 of SBP: SBP ≥ 180 mmHg; M1 of DBP: DBP ≤ 89 mmHg, M2 of DBP: 90 ≤ DBP ≤ 99 mmHg, M3 of DBP: 100 ≤ DBP ≤ 109 mmHg, M4 of DBP: DBP ≥ 110 mmHg. CI, conﬁdence interval; OR, odds ratio; SBP, systole blood pressure; DBP, diastolic blood pressure.

Interaction and joint effects analyses of elevated SBP and DBP and unhealthy lifestyle factors on stroke indicated that unhealthy behaviors and higher blood pressure levels synergistically increased the incidence of stroke after adjusting for confounding factors. Compared with non-smokers with SBP < 140 mmHg or DBP < 90 mmHg, smokers with SBP 140–159 mmHg, 160–179 mmHg, or DBP 100–109 mmHg and ≥110 mmHg had increased stroke incidence by 46%,108%, and 212%, 222%, respectively. Compared with non-drinkers with SBP < 140 mmHg or DBP < 90 mmHg, drinkers with SBP 160–179 mmHg or DBP 100–109 mmHg showed increased stroke incidence by 95% and 157%, respectively. Compared with non-obese individuals with SBP < 140 mmHg or DBP < 90 mmHg, obese participants with SBP 160–179 mmHg, ≥ 180 mmHg, or DBP 100–109 mmHg had increased stroke incidence by 88%, 106%, and 96%, respectively. Compared with physically active individuals with SBP < 140 mmHg or DBP < 90 mmHg, those who were physically inactive with SBP ≥ 180 mmHg or DBP ≥ 110 mmHg exhibited increased stroke incidence by 56% and 115%, respectively (Fig 3).

**Fig 4:**
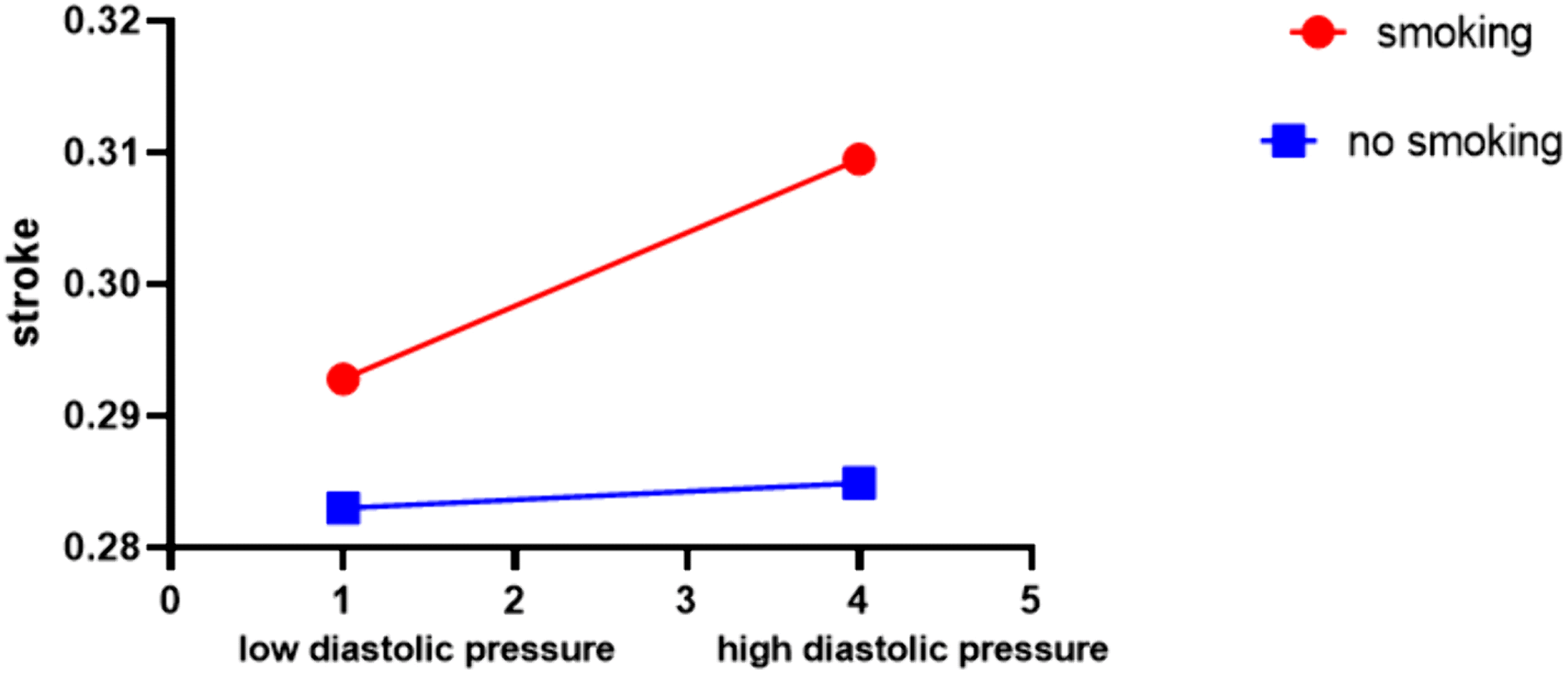
Modulating effect of smoking on diastolic blood pressure. Models were adjusted for sex, age, occupation, marital status, education level, medical insurance, atrial fibrillation, family history of stroke, diabetes, dyslipidemia, and fatty liver.

No significant interactions between systolic blood pressure and stroke incidence were observed across the lifestyle subgroups (P for interaction >0.05)(Figs 1–2). In contrast, the association between diastolic blood pressure and stroke was modified significantly only by smoking status (P for interaction = 0.031). Simple effect analysis further revealed a stronger positive association between DBP and stroke among smokers compared to non-smokers (Fig 4).

## Discussion

This study examined the joint predictive value of SBP, DBP, and lifestyle on incident stroke among adults aged over 65 in northern China. The main findings can be summarized as follows: (1) Based on blood pressure group analyses, higher SBP and DBP were significantly associated with an increased incidence of stroke. This association exhibited a linear trend and remained consistent across most lifestyle subgroups. (2) Unhealthy lifestyle factors synergistically interacted with elevated SBP and DBP levels to further increase the incidence of stroke among older adults. (3) The predictive effect of DBP on stroke incidence was modified by smoking status, indicating a stronger positive association between DBP and stroke among smokers compared to non-smokers.

Among the various risk factors contributing to stroke, hypertension remains a well-established modifiable element, with a global prevalence that warrants significant attention [18]. Our study identified a positive association between blood pressure and the incidence of stroke among adults over 65 years of age. This association remained consistent across subgroups with different lifestyle factors, including smoking, alcohol consumption, physical inactivity, and obesity. These findings align with previous research. For instance, Perry et al. reported that both systolic blood pressure (SBP) and diastolic blood pressure (DBP), whether considered individually or in combination, are independently, continuously, and positively associated with an increased risk of stroke [19]. Similarly, Lewington et al. demonstrated that stroke risk increases linearly once blood pressure levels exceed 115/75 mmHg [8].

Previous studies have also examined the relationship between blood pressure and stroke incidence using stratified analyses, though differences exist in both blood pressure categorization and study populations compared to our research [20]. For example, a survey conducted among elderly Ghanaians calculated incident stroke over an 18-month follow-up using three blood pressure cut-offs: < 120/80 mmHg, 120–159/80–99 mmHg, and ≥160/100 mmHg. This study found that a lower blood pressure target of <120/80 mmHg was associated with a signal of reduced incident stroke [21]. However, although lowering blood pressure generally decreases stroke risk in a linear manner within the general population, a J-shaped relationship has been observed in individuals with diabetes [20,22]. Elevated systolic and diastolic blood pressure can contribute to cerebrovascular diseases through mechanisms such as atherosclerotic plaque formation, smooth muscle cell remodeling, reduced cerebral blood flow, and arterial baroreflex dysfunction [23,24]. Effective antihypertensive treatment has been shown to reduce both the incidence and recurrence risk of stroke [23]. Therefore, active management of blood pressure in hypertensive patients is of considerable importance for stroke risk reduction.

Although the 2017 AHA/ACC guidelines lowered the diagnostic threshold for hypertension from ≥140/90 mmHg to ≥130/80 mmHg in the general population, the 2014 evidence-based guidelines for hypertension in adults have continued to be widely adopted in China [25]. In accordance with these Chinese guidelines, we categorized and analyzed the relationship between systolic and diastolic blood pressure and stroke. This classification approach has also been employed in other similar studies [26,27]. The use of this grouping method provides a valuable reference for future hierarchical management of hypertension. Furthermore, exploring the association between systolic and diastolic blood pressure and stroke based on this classification system offers practical insights for risk-stratified management of hypertension and stroke prevention strategies.

The findings of this study provide substantial evidence for a synergistic effect between lifestyle factors and elevated systolic and diastolic blood pressure on incident stroke. Both unhealthy lifestyles and increased blood pressure are well-established risk factors for stroke, and their individual associations with stroke have been extensively documented [28–34]. This study further demonstrates that when these factors coexist, their combined impact on stroke risk exceeds the sum of their individual effects. This insight suggests that a coordinated strategy targeting both types of risk factors could yield additional benefits in stroke prevention.

The mechanism underlying the interaction between lifestyle and blood pressure in relation to stroke is complex. First, elevated blood pressure is directly associated with an increased risk of stroke. Second, lifestyle factors may exert specific regulatory effects on blood pressure [35–37]. For example, alcohol consumption can raise blood pressure through activation of the sympathetic nervous system [36], and is positively correlated with hypertension risk. The adverse effects of alcohol on blood pressure may also directly increase the risk of hemorrhagic stroke [38]. The relationship between obesity and blood pressure is nearly linear [39], and obesity-related hypertension can further elevate stroke risk. Cigarette smoking, whether active or passive, significantly impairs endothelial function and increases the risk of atherosclerotic disease and hypertension [40]. Furthermore, interventions such as physical activity have been shown to mitigate these effects. For instance, a study of 50 participants with treatment-resistant hypertension reported that 8–12 weeks of aerobic exercise reduced daytime systolic and diastolic ambulatory blood pressure by 5.9 mmHg and 3.3 mmHg, respectively [41]. Thus, unhealthy lifestyles may elevate stroke risk partly through their impact on increasing blood pressure.

In our study, drinkers with elevated SBP and DBP showed a significantly increased incidence of stroke. This finding is consistent with a Korean cohort study, which demonstrated through combined risk analysis that alcohol consumption poses a greater danger for individuals with severe hypertension [38]. Therefore, controlling alcohol intake is particularly important among patients with severe hypertension. However, a prospective population-based cohort study conducted in China indicated that hypertension is an independent risk factor for stroke, while alcohol consumption was not significantly associated in their model [42]. Some studies have even suggested that moderate alcohol consumption may be associated with a reduced risk of ischemic stroke. Proposed mechanisms for such protective effects include cardioprotective properties, antithrombotic effects, and stress reduction [43,44]. The extent to which alcohol-induced hypertension contributes to stroke risk, and the underlying mechanisms, remain incompletely understood. Some evidence suggests that alcohol-related hypertension may involve catecholamine activity and genetically influenced inactivation pathways [45]. Heavy alcohol consumption may elevate blood pressure through activation of the sympathetic nervous system, a mechanism whose sensitivity may be genetically modulated. For instance, the methyl-adenosine phosphorylase rs10118757 G allele has been observed to modify the risk of ischemic stroke related to alcohol-induced hypertension [46]. These findings highlight the potential essential role of genetic factors in alcohol-induced hypertension and associated stroke.

Previous studies have indicated that the effect of physical exercise on blood pressure is influenced by the baseline blood pressure level of the patients. In individuals with stage II and III hypertension, physical exercise may partially substitute for pharmacological therapy by contributing to blood pressure reduction and improving circulatory function [37,47,48]. In contrast, among patients with mild hypertension, the antihypertensive effect of physical exercise has not been consistently significant [49]. Exercise can attenuate the activity of the renin-angiotensin system—a hormone system that promotes vasoconstriction and elevates blood pressure. It has also been shown to enhance endothelial function, which is critical for maintaining vascular tone and stable blood pressure levels [50,51]. Alternatively, the association between physical activity and reduced stroke risk may be attributed to non-blood-pressure-related effects, such as improvements in insulin sensitivity, lipid metabolism, endothelial function, and immune regulation [52].

Obesity is a significant risk factor for hypertension. The Framingham Study, one of the most influential cohort studies, reported that the prevalence of hypertension among obese individuals is twice that of non-obese individuals [53]. The Trials of Hypertension Prevention (TOHP) study demonstrated that for every 2 kg of weight loss, systolic and diastolic blood pressure decreased by 3.7 mmHg and 2.7 mmHg, respectively [54]. Additionally, body mass index (BMI) has been consistently shown to be positively correlated with stroke risk [55]. Animal experiments conducted by Osmond et al. revealed structural remodeling of the middle cerebral artery in obese rats, which exhibited more severe brain damage following cerebral ischemia compared to non-obese controls [56]. These cerebrovascular alterations are associated with the development of hypertension and suggest that elevated blood pressure may be a key mediator of stroke risk in obese individuals. Extensive studies have indicated that obesity, particularly visceral adipose tissue, substantially contributes to increased blood pressure [57]. The mechanisms underlying obesity-related hypertension are multifactorial and include sodium retention, insulin resistance, activation of the renin-angiotensin-aldosterone system, altered vascular function, and dysregulation of adipokine secretion [58,59]. Visceral fat plays a particularly critical role in activating these pathways. These insights underscore the potential benefit of a coordinated approach targeting both obesity and hypertension for enhanced stroke prevention.

A key finding of our study is the significant interaction between smoking status and diastolic blood pressure (DBP) in predicting stroke incidence. This interaction indicates a critical synergistic effect, in which the combined influence of smoking and elevated DBP on stroke risk exceeds the sum of their individual effects [60]. Further analysis confirmed that the positive association between DBP and stroke is substantially stronger among smokers than among non-smokers.

Smoking, a well-established independent risk factor for stroke, adversely affects vascular health through multiple mechanisms, including endothelial dysfunction, accelerated atherosclerosis, increased blood viscosity, and prothrombotic states [61–64]. Elevated DBP, on the other hand, contributes to chronic vascular stress, impairs microvascular perfusion, and increases the risk of cerebral small vessel disease [65–68]—each of which represents a key pathway in stroke pathogenesis. Our findings suggest that these two factors may act synergistically to amplify stroke risk: smoking may intensify the vascular damage associated with elevated DBP, or high DBP may exacerbate the prothrombotic and atherosclerotic effects of smoking. This mutual reinforcement could explain why the association between DBP and stroke is magnified among smokers, underscoring the importance of evaluating their combined impact in clinical risk assessment [5]. Notably, this interaction was specific to DBP, as no significant interactions were observed between systolic blood pressure (SBP) and smoking or other lifestyle factors in our analyses. This divergence raises important questions regarding the distinct roles of SBP and DBP in stroke pathogenesis, particularly under the influence of smoking. While SBP is widely regarded as a primary driver of macrovascular events, DBP may play a more critical role in microvascular injury—a process potentially aggravated by smoking-related endothelial damage. This specificity warrants further investigation to elucidate the underlying physiological mechanisms.

From a clinical perspective, our findings highlight the need for tailored risk management strategies. The significant interaction between smoking and DBP identifies a high-risk subgroup—smokers with elevated DBP—for whom targeted interventions could substantially reduce stroke incidence. These results enhance our understanding of how lifestyle factors and physiological markers interact to shape cardiovascular risk, with important implications for clinical practice and public health policy.

### Limitation

The strengths of this study include its large sample size (n = 34,995) and the examination of interactive effects between systolic and diastolic blood pressure and lifestyle factors on stroke. However, several limitations should be acknowledged. First, due to the observational design, causality cannot be established, and the possibility of reverse causality cannot be excluded. Second, although our models adjusted for numerous covariates, residual confounding from unmeasured variables—such as diet, sleep quality, and inflammatory markers—may still remain, a challenge common to observational studies. Third, lifestyle factors were self-reported via a structured questionnaire, which may introduce recall bias and potential misclassification. Additionally, due to limitations in the questionnaire design, only leisure-time/recreational and occupational physical activity were assessed; transport-related physical activity was not evaluated. Thus, the findings should be interpreted with caution within these constraints. Finally, the study population was drawn from a single province in northern China. While this sample may be partially representative of the broader northern Chinese population, it does not fully capture the region’s diversity. Therefore, further validation through multi-regional, population-based longitudinal studies involving more diverse demographics is warranted.

## Conclusion

The observed synergistic effect between blood pressure (systolic and diastolic) and lifestyle factors, along with the interaction effect of lifestyle in the blood pressure–stroke relationship, has important implications for stroke prevention and management. First, primary prevention strategies should emphasize reducing unhealthy lifestyle behaviors and controlling hypertension. Second, healthcare providers should assess both traditional risk factors and the interaction between blood pressure and lifestyle when evaluating stroke risk. Hypertension patients, in particular, require intensified lifestyle management. Integrating blood pressure and lifestyle profiles into existing risk assessment tools could improve risk stratification and help identify high-risk individuals for early intervention. Third, these findings underscore the value of individualized treatment. Clinicians should collaborate with patients to develop personalized plans that combine pharmacological treatments with lifestyle modifications tailored to specific risk profiles.

## Supporting information

S1 Table Clinical characteristics of person according to the specified Systolic and diastolic pressure categories. (DOCX)

S2 Table Subgroup analyses among the group M1 − 4 of DBP and Stroke across various lifestyle subgroups. (DOCX)

S3 Table Subgroup analyses among the group Q1 − 4 of SBP and Stroke across various lifestyle subgroups. (DOCX)

## References

[1] Pu L, Wang L, Zhang R, Zhao T, Jiang Y, Han L. Projected global trends in ischemic stroke incidence, deaths and disability-adjusted life years from 2020 to 2030. Stroke. 2023;54(5):1330–9. doi: 10.1161/STROKEAHA.122.040073 37094034

[2] GBD 2016 Lifetime Risk of Stroke Collaborators, Feigin VL, Nguyen G, Cercy K, Johnson CO, Alam T, et al. Global, Regional, and Country-Specific Lifetime Risks of Stroke, 1990 and 2016. N Engl J Med. 2018;379(25):2429–37. doi: 10.1056/NEJMoa1804492 30575491 PMC6247346

[3] Wang W, Jiang B, Sun H, Ru X, Sun D, Wang L, et al. Prevalence, incidence, and mortality of stroke in china: results from a nationwide population-based survey of 480 687 adults. Circulation. 2017;135(8):759–71. doi: 10.1161/CIRCULATIONAHA.116.025250 28052979

[4] Chen J, Zhu Q, Yu L, Li Y, Jia S, Zhang J. Stroke risk factors of stroke patients in China: A Nationwide Community-Based Cross-Sectional Study. Int J Environ Res Public Health. 2022;19(8):4807. doi: 10.3390/ijerph19084807 35457673 PMC9030671

[5] Lee SM, Oh C-M, Kim M-H, Ha E, Hong M, Ryoo J-H. Current smoking status as a predictor of cerebral infarction in men: a retrospective cohort study in South Korea. BMJ Open. 2021;11(4):e042317. doi: 10.1136/bmjopen-2020-042317 33853795 PMC8054067

[6] Setyopranoto I, Bayuangga HF, Panggabean AS, Alifaningdyah S, Lazuardi L, Dewi FST, et al. Prevalence of stroke and associated risk factors in sleman district of yogyakarta special region, Indonesia. Stroke Res Treat. 2019;2019:2642458. doi: 10.1155/2019/2642458 31186829 PMC6521526

[7] O’Donnell MJ, Chin SL, Rangarajan S, Xavier D, Liu L, Zhang H, et al. Global and regional effects of potentially modifiable risk factors associated with acute stroke in 32 countries (INTERSTROKE): a case-control study. Lancet. 2016;388(10046):761–75. doi: 10.1016/S0140-6736(16)30506-2 27431356

[8] Lewington S, Clarke R, Qizilbash N, Peto R, Collins R, Prospective Studies Collaboration. Age-specific relevance of usual blood pressure to vascular mortality: a meta-analysis of individual data for one million adults in 61 prospective studies. Lancet. 2002;360(9349):1903–13. doi: 10.1016/s0140-6736(02)11911-8 12493255

[9] Miura K, Soyama Y, Morikawa Y, Nishijo M, Nakanishi Y, Naruse Y, et al. Comparison of four blood pressure indexes for the prediction of 10-year stroke risk in middle-aged and older Asians. Hypertension. 2004;44(5):715–20. doi: 10.1161/01.HYP.0000145108.23948.7b 15452026

[10] Makino Y, Kawano Y, Minami J, Yamaguchi T, Takishita S. Risk of stroke in relation to level of blood pressure and other risk factors in treated hypertensive patients. Stroke. 2000;31(1):48–52. doi: 10.1161/01.str.31.1.48 10625714

[11] Ovbiagele B. Low-normal systolic blood pressure and secondary stroke risk. J Stroke Cerebrovasc Dis. 2013;22(5):633–8. doi: 10.1016/j.jstrokecerebrovasdis.2011.12.003 22244715

[12] Lyden P, Schwamm LH, Mohl SM. 2022 Update From the American Stroke Association and the Stroke Council. Stroke. 2022;53(8):e383–4. doi: 10.1161/STROKEAHA.122.039161 35877821

[13] Mancia G, Kreutz R, Brunström M, Burnier M, Grassi G, Januszewicz A, et al. 2023 ESH guidelines for the management of arterial hypertension the task force for the management of arterial hypertension of the european society of hypertension: endorsed by the International Society of Hypertension (ISH) and the European Renal Association (ERA). J Hypertens. 2023;41(12):1874–2071. doi: 10.1097/HJH.0000000000003480 37345492

[14] Wang J-G, Zhang W, Li Y, Liu L. Hypertension in China: epidemiology and treatment initiatives. Nat Rev Cardiol. 2023;20(8):531–45. doi: 10.1038/s41569-022-00829-z 36631532

[15] Yu J, Yi Q, Chen G, Hou L, Liu Q, Xu Y, et al. The visceral adiposity index and risk of type 2 diabetes mellitus in China: A national cohort analysis. Diabetes Metab Res Rev. 2022;38(3):e3507. doi: 10.1002/dmrr.3507 34679251

[16] Karr S. Epidemiology and management of hyperlipidemia. Am J Manag Care. 2017;23(9 Suppl):S139–48. 28978219

[17] Weir CB, Jan A. BMI classification percentile and cut off points. StatPearls. Treasure Island (FL): StatPearls Publishing. 2023.

[18] Furie K. Epidemiology and primary prevention of stroke. Continuum (Minneap Minn). 2020;26(2):260–7. doi: 10.1212/CON.0000000000000831 32224751

[19] Perry HM Jr, Davis BR, Price TR, Applegate WB, Fields WS, Guralnik JM, et al. Effect of treating isolated systolic hypertension on the risk of developing various types and subtypes of stroke: the Systolic Hypertension in the Elderly Program (SHEP). JAMA. 2000;284(4):465–71. doi: 10.1001/jama.284.4.465 10904510

[20] Weber MA, Bloch M, Bakris GL, Weir MR, Zappe DH, Dahlof B, et al. Cardiovascular Outcomes According to Systolic Blood Pressure in Patients With and Without Diabetes: An ACCOMPLISH Substudy. J Clin Hypertens (Greenwich). 2016;18(4):299–307. doi: 10.1111/jch.12816 27060568 PMC8032014

[21] Sarfo FS, Mobula LM, Adade T, Commodore-Mensah Y, Agyei M, Kokuro C, et al. Low blood pressure levels & incident stroke risk among elderly Ghanaians with hypertension. J Neurol Sci. 2020;413:116770. doi: 10.1016/j.jns.2020.116770 32172015 PMC7250714

[22] Zhao W, Katzmarzyk PT, Horswell R, Wang Y, Johnson J, Cefalu WT, et al. Blood pressure and stroke risk among diabetic patients. J Clin Endocrinol Metab. 2013;98(9):3653–62. doi: 10.1210/jc.2013-1757 23714680 PMC5393468

[23] SPRINT MIND Investigators for the SPRINT Research Group, Williamson JD, Pajewski NM, Auchus AP, Bryan RN, Chelune G, et al. Effect of intensive vs standard blood pressure control on probable dementia: a randomized clinical trial. JAMA. 2019;321(6):553–61. doi: 10.1001/jama.2018.21442 30688979 PMC6439590

[24] Setyopranoto I, Bayuangga HF, Panggabean AS, Alifaningdyah S, Lazuardi L, Dewi FST, et al. Prevalence of Stroke and Associated Risk Factors in Sleman District of Yogyakarta Special Region, Indonesia. Stroke Res Treat. 2019;2019:2642458. doi: 10.1155/2019/2642458 31186829 PMC6521526

[25] James PA, Oparil S, Carter BL, Cushman WC, Dennison-Himmelfarb C, Handler J, et al. 2014 evidence-based guideline for the management of high blood pressure in adults: report from the panel members appointed to the Eighth Joint National Committee (JNC 8). JAMA. 2014;311(5):507–20. doi: 10.1001/jama.2013.284427 24352797

[26] Nordahl H, Osler M, Frederiksen BL, Andersen I, Prescott E, Overvad K, et al. Combined effects of socioeconomic position, smoking, and hypertension on risk of ischemic and hemorrhagic stroke. Stroke. 2014;45(9):2582–7. doi: 10.1161/STROKEAHA.114.005252 25123220

[27] Ge Z, Hao Y, Cao J, Li J, Chen J, Huang J, et al. Does cigarette smoking exacerbate the effect of blood pressure on the risk of cardiovascular and all-cause mortality among hypertensive patients?. J Hypertens. 2012;30(12):2307–13. doi: 10.1097/HJH.0b013e328359aa1f 23032144

[28] Gan Y, Feng J, Zhu Y, Li L, Shen X, Lou Y, et al. Association between alcohol consumption and the risk of stroke in middle-aged and older adults in China. Drug Alcohol Depend. 2021;229(Pt B):109134. doi: 10.1016/j.drugalcdep.2021.109134 34847483

[29] Yang W, Kang D-W, Ha SY, Lee S-H. Drinking Patterns and Risk of Ischemic Stroke in Middle-Aged Adults: Do Beneficial Drinking Habits Indeed Exist?. Stroke. 2021;52(1):164–71. doi: 10.1161/STROKEAHA.120.032144 33148143

[30] Forlivesi S, Cappellari M, Bonetti B. Obesity paradox and stroke: a narrative review. Eat Weight Disord. 2021;26(2):417–23. doi: 10.1007/s40519-020-00876-w 32124408

[31] Zhang P, Yan X-L, Qu Y, Guo Z-N, Yang Y. Association between abnormal body weight and stroke outcome: A meta-analysis and systematic review. Eur J Neurol. 2021;28(8):2552–64. doi: 10.1111/ene.14881 33896081

[32] García-Cabo C, López-Cancio E. Exercise and stroke. Adv Exp Med Biol. 2020;1228:195–203. doi: 10.1007/978-981-15-1792-1_13 32342459

[33] Tran P, Tran L, Tran L. Smoking levels and associations between sociodemographic factors and smoking continuation in U.S. stroke survivors. Ann Epidemiol. 2020;43:66–70. doi: 10.1016/j.annepidem.2020.01.007 32094041

[34] Xu L, Schooling CM, Chan WM, Lee SY, Leung GM, Lam TH. Smoking and hemorrhagic stroke mortality in a prospective cohort study of older Chinese. Stroke. 2013;44(8):2144–9. doi: 10.1161/STROKEAHA.113.001500 23723306

[35] Okubo Y, Sairenchi T, Irie F, Yamagishi K, Iso H, Watanabe H, et al. Association of alcohol consumption with incident hypertension among middle-aged and older Japanese population: the Ibarakai Prefectural Health Study (IPHS). Hypertension. 2014;63(1):41–7. doi: 10.1161/HYPERTENSIONAHA.113.01585 24126168

[36] Ikehara S, Iso H. Alcohol consumption and risks of hypertension and cardiovascular disease in Japanese men and women. Hypertens Res. 2020;43(6):477–81. doi: 10.1038/s41440-020-0417-1 32203447

[37] Alpsoy Ş. Exercise and Hypertension. Adv Exp Med Biol. 2020;1228:153–67. doi: 10.1007/978-981-15-1792-1_10 32342456

[38] Sull JW, Yi SW, Nam CM, Choi K, Ohrr H. Binge drinking and hypertension on cardiovascular disease mortality in Korean men and women: a Kangwha cohort study. Stroke. 2010;41(10):2157–62. doi: 10.1161/STROKEAHA.110.586347 20724719

[39] Fauchier G, Bisson A, Bodin A, Herbert J, Semaan C, Angoulvant D, et al. Metabolically healthy obesity and cardiovascular events: A nationwide cohort study. Diabetes Obes Metab. 2021;23(11):2492–501. doi: 10.1111/dom.14492 34251088

[40] Wang J-G, Zhang W, Li Y, Liu L. Hypertension in China: epidemiology and treatment initiatives. Nat Rev Cardiol. 2023;20(8):531–45. doi: 10.1038/s41569-022-00829-z 36631532

[41] Dimeo F, Pagonas N, Seibert F, Arndt R, Zidek W, Westhoff TH. Aerobic exercise reduces blood pressure in resistant hypertension. Hypertension. 2012;60(3):653–8. doi: 10.1161/HYPERTENSIONAHA.112.197780 22802220

[42] Tang L, Xu T, Li H, Zhang M, Wang A, Tong W, et al. Hypertension, alcohol drinking and stroke incidence: a population-based prospective cohort study among inner mongolians in China. J Hypertens. 2014;32(5):1091–6; discussion 1096. doi: 10.1097/HJH.0000000000000142 24577411

[43] Mostofsky E, Chahal HS, Mukamal KJ, Rimm EB, Mittleman MA. Alcohol and immediate risk of cardiovascular events: a systematic review and dose-response meta-analysis. Circulation. 2016;133(10):979–87. doi: 10.1161/CIRCULATIONAHA.115.019743 26936862 PMC4783255

[44] Hillbom M, Juvela S, Numminen H. Alcohol intake and the risk of stroke. J Cardiovasc Risk. 1999;6(4):223–8. doi: 10.1177/204748739900600406 10501273

[45] Stewart SH, Oroszi G, Randall PK, Anton RF. COMT genotype influences the effect of alcohol on blood pressure: results from the COMBINE study. Am J Hypertens. 2009;22(1):87–91. doi: 10.1038/ajh.2008.321 19023276 PMC2630169

[46] Li S, Hu W, Liu D, Sun F, Zhang Q, Yang X, et al. MTAP gene is associated with ischemic stroke in Chinese Hans. J Neurol Sci. 2009;284(1–2):103–7. doi: 10.1016/j.jns.2009.04.013 19427650

[47] Nascimento LS, Santos AC, Lucena J, Silva L, Almeida A, Brasileiro-Santos MS. Acute and chronic effects of aerobic exercise on blood pressure in resistant hypertension: study protocol for a randomized controlled trial. Trials. 2017;18(1):250. doi: 10.1186/s13063-017-1985-5 28578691 PMC5457580

[48] Lopes S, Afreixo V, Teixeira M, Garcia C, Leitão C, Gouveia M, et al. Exercise training reduces arterial stiffness in adults with hypertension: a systematic review and meta-analysis. J Hypertens. 2021;39(2):214–22. doi: 10.1097/HJH.0000000000002619 32833924

[49] Blumenthal JA, Siegel WC, Appelbaum M. Failure of exercise to reduce blood pressure in patients with mild hypertension. Results of a randomized controlled trial. JAMA. 1991;266(15):2098–104. doi: 10.1001/jama.1991.03470150070033 1920698

[50] Hannan M, Kringle E, Hwang C-L, Laddu D. Behavioral medicine for sedentary behavior, daily physical activity, and exercise to prevent cardiovascular disease: a review. Curr Atheroscler Rep. 2021;23(9):48. doi: 10.1007/s11883-021-00948-x 34226989 PMC8257263

[51] Strambi M, Giussani M, Ambruzzi MA, Brambilla P, Corrado C, Giordano U, et al. Novelty in hypertension in children and adolescents: focus on hypertension during the first year of life, use and interpretation of ambulatory blood pressure monitoring, role of physical activity in prevention and treatment, simple carbohydrates and uric acid as risk factors. Ital J Pediatr. 2016;42(1):69. doi: 10.1186/s13052-016-0277-0 27423331 PMC4947361

[52] Warburton DER, Nicol CW, Bredin SSD. Health benefits of physical activity: the evidence. CMAJ. 2006;174(6):801–9. doi: 10.1503/cmaj.051351 16534088 PMC1402378

[53] Aronow WS. Association of obesity with hypertension. Ann Transl Med. 2017;5(17):350. doi: 10.21037/atm.2017.06.69 28936444 PMC5599277

[54] The effects of nonpharmacologic interventions on blood pressure of persons with high normal levels. Results of the Trials of Hypertension Prevention, Phase I. JAMA. 1992;267(9):1213–20. doi: 10.1001/jama.1992.03480090061028 1586398

[55] Li W, Katzmarzyk PT, Horswell R, Zhang Y, Zhao W, Wang Y, et al. Body mass index and stroke risk among patients with type 2 diabetes mellitus. Stroke. 2015;46(1):164–9. doi: 10.1161/STROKEAHA.114.006718 25468880 PMC4276457

[56] Osmond JM, Mintz JD, Dalton B, Stepp DW. Obesity increases blood pressure, cerebral vascular remodeling, and severity of stroke in the Zucker rat. Hypertension. 2009;53(2):381–6. doi: 10.1161/HYPERTENSIONAHA.108.124149 19104000 PMC2839545

[57] Stabouli S, Papakatsika S, Kotsis V. The role of obesity, salt and exercise on blood pressure in children and adolescents. Expert Rev Cardiovasc Ther. 2011;9(6):753–61. doi: 10.1586/erc.11.63 21714606

[58] Kotchen TA. Obesity-related hypertension: epidemiology, pathophysiology, and clinical management. Am J Hypertens. 2010;23(11):1170–8. doi: 10.1038/ajh.2010.172 20706196

[59] Boscaro M, Giacchetti G, Ronconi V. Visceral adipose tissue: emerging role of gluco- and mineralocorticoid hormones in the setting of cardiometabolic alterations. Ann N Y Acad Sci. 2012;1264(1):87–102. doi: 10.1111/j.1749-6632.2012.06597.x 22804097 PMC3464353

[60] Shihab S, Boucher RE, Abraham N, Wei G, Beddhu S. Influence of Baseline Diastolic Blood Pressure on the Effects of Intensive Systolic Blood Pressure Lowering on the Risk of Stroke. Hypertension. 2022;79(4):785–93. doi: 10.1161/HYPERTENSIONAHA.121.18172 35114798 PMC8917090

[61] Hajat C, Stein E, Ramstrom L, Shantikumar S, Polosa R. The health impact of smokeless tobacco products: a systematic review. Harm Reduct J. 2021;18(1):123. doi: 10.1186/s12954-021-00557-6 34863207 PMC8643012

[62] Ambrose JA, Barua RS. The pathophysiology of cigarette smoking and cardiovascular disease: an update. J Am Coll Cardiol. 2004;43(10):1731–7. doi: 10.1016/j.jacc.2003.12.047 15145091

[63] Lowery CL 3rd, Woulfe D, Kilic F. Responses of Plasma Catecholamine, Serotonin, and the Platelet Serotonin Transporter to Cigarette Smoking. Front Neurosci. 2019;13:32. doi: 10.3389/fnins.2019.00032 30886568 PMC6409334

[64] Renaud S, Blache D, Dumont E, Thevenon C, Wissendanger T. Platelet function after cigarette smoking in relation to nicotine and carbon monoxide. Clin Pharmacol Ther. 1984;36(3):389–95. doi: 10.1038/clpt.1984.193 6467799

[65] Lee M, Ovbiagele B, Hong K-S, Wu Y-L, Lee J-E, Rao NM, et al. Effect of Blood Pressure Lowering in Early Ischemic Stroke: Meta-Analysis. Stroke. 2015;46(7):1883–9. doi: 10.1161/STROKEAHA.115.009552 26022636

[66] Franklin SS, Gustin W 4th, Wong ND, Larson MG, Weber MA, Kannel WB, et al. Hemodynamic patterns of age-related changes in blood pressure. The Framingham Heart Study. Circulation. 1997;96(1):308–15. doi: 10.1161/01.cir.96.1.308 9236450

[67] Hägg-Holmberg S, Dahlström EH, Forsblom CM, Harjutsalo V, Liebkind R, Putaala J, et al. The role of blood pressure in risk of ischemic and hemorrhagic stroke in type 1 diabetes. Cardiovasc Diabetol. 2019;18(1):88. doi: 10.1186/s12933-019-0891-4 31288813 PMC6617855

[68] Rawshani A, Rawshani A, Sattar N, Franzén S, McGuire DK, Eliasson B, et al. Relative Prognostic Importance and Optimal Levels of Risk Factors for Mortality and Cardiovascular Outcomes in Type 1 Diabetes Mellitus. Circulation. 2019;139(16):1900–12. doi: 10.1161/CIRCULATIONAHA.118.037454 30798638

