# Supplementary material for: The interaction between lifestyle and blood pressure on Stroke: A cross-sectional study from Northern China
Source: PLoS One. 2026 Mar 9;21(3):e0344016. doi: 10.1371/journal.pone.0344016 (PMC12970864; doi:10.1371/journal.pone.0344016)
Supplement: S1 Table — (DOCX) [file pone.0344016.s001.docx]

Table1 Clinical characteristics of person according to the specified Systolic and diastolic pressure categories

|  | systolic pressure mmHg | | | | diastolic pressure mmHg | | | |
| --- | --- | --- | --- | --- | --- | --- | --- | --- |
| Variable N(n%) | <139 | 140-159 | 160-179 | >180 | <89 | 90-99 | 100-109 | >110 |
| Sex n(%) |  |  |  |  |  |  |  |  |
| femal | 13077（55.0） | 4422（56.5） | 1464（58.7） | 548（59.8） | 16749(56.3) | 2147(54.4) | 482(48.7) | 133(46.0) |
| Age n(%) |  |  |  |  |  |  |  |  |
| 64-74 | 17488（73.6） | 5626（71.9） | 1785（71.6） | 618（67.4） | 21603(72.6) | 2951(74.8) | 752(76.0) | 211(73.0) |
| 75-84 | 5380（22.6） | 1962（25.1） | 628（25.2） | 264（38.8） | 7074(23.8) | 883(22.4) | 210(21.2) | 67(23.2) |
| >85 | 892（3.8） | 237（3.0） | 80（3.2） | 35（3.8） | 1093(3.7) | 112(2.8) | 28(2.8) | 11(3.8) |
| Education level n(%) |  |  |  |  |  |  |  |  |
| <HS | 14615（61.5） | 4963（63.4） | 1683（67.5） | 669（73.0） | 18442(61.9) | 2630(66.6) | 654(66.1) | 204(70.6) |
| HS graduate | 8504（35.8） | 2663（34.0） | 764（30.6） | 241（36.3） | 10523(35.3) | 1247(31.6) | 319(32.2) | 83(28.7) |
| College graduate | 641（2.7） | 199（2.5） | 46（1.8） | 7（0.8） | 805(2.7) | 69(1.7) | 17(1.7) | 2(0.7) |
| Marital status n(%) |  |  |  |  |  |  |  |  |
| Married | 475（2.0） | 137（1.8） | 42（1.7） | 20（2.2） | 563（1.9） | 76（1.9） | 29（2.9） | 6（2.1） |
| Never married | 19586（82.4） | 6392（81.7） | 2008（80.5） | 700（76.3） | 24462（82.2） | 3215（81.5） | 781（78.9） | 228（78.9） |
| Widowed/Divorced | 3407（14.3） | 1219（15.6） | 404（16.2） | 175（19.1） | 4383（14.7） | 612（15.5） | 164（16.6） | 46（15.9） |
| others | 292（1.2） | 77（1.0） | 39（1.6） | 22（2.4） | 362（1.2） | 43（1.1） | 16（1.6） | 9（3.1） |
| Occupation n(%) |  |  |  |  |  |  |  |  |
| laborer | 1444（6.1） | 486（6.2） | 129（5.2） | 33（3.6） | 1836(6.2) | 201(5.1) | 46(4.6) | 9(3.1) |
| peasant | 19847（83.5） | 6539（83.6） | 2163（86.8） | 832（90.7） | 24829(83.4) | 3411(86.4) | 870(87.9) | 271(93.8) |
| leadership | 2469（10.4） | 800（10.2） | 201（8.1） | 52（5.7） | 3105(10.4) | 334(8.5) | 74(7.5) | 9(3.1) |
| Medical Insurance(Yes) n(%) | 23256（97.9） | 7653（97.8） | 2418（97） | 879（95.9） | 29129(97.8) | 3842(97.4) | 959(96.9) | 276(95.5) |
| Atrial fibrillation n(%) | 136（0.6） | 37（0.5） | 13（0.5） | 9（1.0） | 146（0.5） | 28（0.7） | 15（1.5） | 6（2.1） |
| Diabetes mellitus n(%) | 5218（22.0） | 2017（25.8） | 685（27.5） | 232（35.3） | 6863(23.1) | 1017(25.8) | 212(21.4) | 60(20.8) |
| Family history of stroke n(%) | 170（0.7） | 59（0.8） | 17（0.7） | 10（1.1） | 227(0.8) | 21(0.5) | 4(0.4) | 4(1.4) |
| besity n(%) | 2851（12.0） | 1421（18.2） | 524（21.0） | 189（20.6） | 3989（13.4） | 737（18.7） | 198（20.0） | 61（21.1） |
| Smoke n(%) | 2548（10.7） | 1018（13.0） | 320（12.8） | 125（13.6） | 3311（11.1） | 509（12.9） | 133（13.4） | 58（20.1） |
| Drink n(%) | 2237（9.4） | 929（11.9） | 320（12.8） | 118（12.9） | 2895（9.7） | 500（12.7） | 151（15.3） | 58（20.1） |
| No Exercise n(%) | 9777（41.1） | 3038（38.8） | 864（34.7） | 307（33.5） | 12224（41.1） | 1374（34.8） | 303（30.6） | 85（29.4） |
