# Supplementary material for: The interaction between lifestyle and blood pressure on Stroke: A cross-sectional study from Northern China
Source: PLoS One. 2026 Mar 9;21(3):e0344016. doi: 10.1371/journal.pone.0344016 (PMC12970864; doi:10.1371/journal.pone.0344016)
Supplement: S2 Table — (DOCX) [file pone.0344016.s002.docx]

Table 2 Subgroup analyses among the group M1−4 of DBP and Stroke across various lifestyle subgroups.

| Lifestyle | diastolic pressure, mmHg | | | | |
| --- | --- | --- | --- | --- | --- |
|  | M1 | M2 | M3 | M4 | *P for trend* |
|  | ≤ 89 | 90-99 | 100-109 | ≥ 110 |  |
| Smoking |  |  |  |  |  |
| yes | 1 | 1.223(0.758-1.974) | 2.693(1.403-5.169) | 2.938(1.131-7.630) | 0.001 |
| no | 1 | 0.970(0.770-1.221) | 1.229(0.824-1.833) | 1.521(0.775-2.987) | 0.286 |
| Drinking |  |  |  |  |  |
| yes | 1 | 0.985(0.563-1.723) | 2.565(1.327-4.958) | 1.848(0.559-6.104) | 0.030 |
| no | 1 | 1.018(0.814-1.274) | 1.246(0.836-1.857) | 1.822(0.985-3.370) | 0.078 |
| Exercise |  |  |  |  |  |
| yes | 1 | 1.042(0.757-1.433) | 1.394(0.790-2.46) | 0.784(0.192-3.208) | 0.531 |
| no | 1 | 1.024(0.779-1.347) | 1.569(1.030-2.393) | 2.468(1.357-4.487) | 0.003 |
| Obesity |  |  |  |  |  |
| yes | 1 | 0.717(0.433-1.186) | 1.639(0.842-3.194) | 1.758(0.614-5.033) | 0.027 |
| no | 1 | 1.084(0.863-1.362) | 1.382(0.933-2.049) | 1.719(0.904-3.266) | 0.394 |

The model adjusted for sex, age, occupation, marital status, education level, health insurance, atrial fibrillation, family history of stroke, diabetes, dyslipidemia, and fatty liver *P < 0.05, **P < 0.01, ***P < 0.001.
