# Supplementary material for: The interaction between lifestyle and blood pressure on Stroke: A cross-sectional study from Northern China
Source: PLoS One. 2026 Mar 9;21(3):e0344016. doi: 10.1371/journal.pone.0344016 (PMC12970864; doi:10.1371/journal.pone.0344016)
Supplement: S3 Table — (DOCX) [file pone.0344016.s003.docx]

Table 3 Subgroup analyses among the group Q1−4 of SBP and Stroke across various lifestyle subgroups

| Lifestyle | Systolic pressure, mmHg | | | |  |
| --- | --- | --- | --- | --- | --- |
|  | Q1 | Q2 | Q3 | Q4 | *P for trend* |
|  | ≤ 139 | 140-159 | 160-179 | ≥ 180 |  |
| Smoking |  |  |  |  |  |
| yes | 1 | 1.210(0.828-1.769) | 1.768(1.054-2.965) | 1.452(0.615-3.429) | 0.041 |
| no | 1 | 1.021(0.855-1.218) | 1.253(0.964-1.628) | 1.598(1.102-2.319) | 0.010 |
| Drinking |  |  |  |  |  |
| yes | 1 | 1.171(0.758-1.809) | 1.937(1.126-3.333) | 1.193(0.422-3.368) | 0.071 |
| no | 1 | 1.046(0.880-1.243) | 1.247(0.962-1.616) | 1.658(1.155-2.381) | 0.005 |
| Exercise |  |  |  |  |  |
| yes | 1 | 0.936(0.734-1.195) | 1.252(0.866-1.809) | 1.256(0.695-2.269) | 0.342 |
| no | 1 | 1.203(0.972-1.490) | 1.481(1.095-2.002) | 1.902(1.250-2.894) | 0.001 |
| Obesity |  |  |  |  |  |
| yes | 1 | 1.139(0.788-1.647) | 1.616(1.010-2.587) | 1.774(0.894-3.517) | 0.019 |
| no | 1 | 1.031(0.862-1.233) | 1.247(0.951-1.636) | 1.499(1.009-2.229) | 0.023 |

The model adjusted for sex, age, occupation, marital status, education level, health insurance, atrial fibrillation, family history of stroke, diabetes, dyslipidemia, and fatty liver *P < 0.05, **P < 0.01, ***P < 0.001.
